# Glucose metabolism sustains heme-induced *Trypanosoma cruzi* epimastigote growth *in vitro*

**DOI:** 10.1371/journal.pntd.0011725

**Published:** 2023-11-10

**Authors:** Carolina Silva Dias Vieira, Ramon Pinheiro Aguiar, Natalia Pereira de Almeida Nogueira, Gilson Costa dos Santos Junior, Marcia Cristina Paes

**Affiliations:** 1 Laboratório de Interação Tripanossomatídeos e Vetores—Departamento de Bioquímica, IBRAG–UERJ–Rio de Janeiro, Brazil; 2 Institute of Medical Biochemistry Leopoldo de Meis (IBqM) and National Center for Structural Biology and Bioimaging (CENABIO)–UFRJ–Rio de Janeiro, Brazil; 3 Instituto Nacional de Ciência e Tecnologia—Entomologia Molecular (INCT-EM)–Brazil; 4 Laboratory of NMR Metabolomics—Departamento de Genética–IBRAG–UERJ–Rio de janeiro, Brazil; US Food and Drug Administration, UNITED STATES

## Abstract

Chagas disease is caused by the protozoan parasite, *Trypanosoma cruzi*. This parasite alternates between an insect vector and a mammalian host. *T*. *cruzi* epimastigotes reside in the insect vector and coexist with the blood components of the vertebrate host. The metabolic profile of *T*. *cruzi* has been extensively studied; however, changes in its metabolism in response to signaling molecules present in the vector are poorly understood. Heme acts as a physiological oxidant that triggers intense epimastigote proliferation and upregulates the expression of genes related to glycolysis and aerobic fermentation *in vitro*. Here, heme-cultured epimastigotes increased D-glucose consumption. In fact, heme-cultured parasites secreted more succinate (the end product of the so-called succinic fermentation) followed by glucose intake. Increased succinate levels reduced the extracellular pH, leading to acidification of the supernatant. However, the acidification and proliferation stimulated by heme was impaired when glycolysis was inhibited. Otherwise, when glucose amount is enhanced in supernatant, heme-cultured parasites increased its growth whereas the glucose depletion caused a delay in proliferation. Heme supplementation increased epimastigote electron transport system-related O_2_ consumption rates, while glucose addition reduced both the electron transport system-related O_2_ consumption rates and spare respiratory capacity, indicating a Crabtree-*like* effect. These results show that glycolysis predominated in heme-cultured epimastigotes over oxidative phosphorylation for energy supply when glucose is present to sustain its high proliferation *in vitro*. Furthermore, it provided an insight into the parasite biology in the vector environment that supply glucose and the digestion of blood generates free heme that can lead to the growth of *T*. *cruzi* epimastigotes.

## Author summary

*Trypanosoma cruzi* is a protozoan parasite causing Chagas disease in humans. Epimastigote forms of the parasite must cope with different challenges, including redox imbalance and nutrient availability. Epimastigotes proliferate inside the insect vector in the presence of heme, a byproduct of blood digestion. This can upregulate the expression of genes related to glycolysis and aerobic fermentation in these parasites. This work shows that heme-cultured epimastigotes enhanced D-glucose consumption, with elevated succinate production and secretion into the culture medium leading to pH acidification, that was blocked by glycolysis inhibitor. Heme increased the maximum oxygen consumption rates, however, the oxygen consumption rate immediately decreased after D-glucose addition in a Crabtree-*like* effect. Interrupting the glycolytic pathway does not result in the increase promoted by heme. Therefore, the growth signaled by heme depends on D-glucose.

## Introduction

Chagas disease (CD) is a potentially life-threatening illness caused by the flagellate protozoan, *Trypanosoma cruzi* [1]. This neglected illness affects seven to eight million people worldwide, mostly people in endemic areas such as Latin America [2]. *The classic T*. *cruzi transmission occurs* through contact with feces of the infected insect vector triatomine during feeding on mammalian host blood.

*T*. *cruzi* is challenged by various factors during its journey inside the insect and mammalian host, including different carbon source availability in each environment. Epimastigotes are the proliferative form of *T*. *cruzi* that can catabolize D-glucose (Glc), amino acids, and lipids from the environment; however, glucose is preferred for proliferating epimastigotes *in vitro* [3–6]. For energy requirement, glucose is partially oxidized in still reduced compounds which are secreted in the medium. This process is called aerobic fermentation and occurs inside a peroxisome-related specialized organelle, the glycosome. The depletion of glucose causes epimastigote metabolism to shift to amino acid consumption for energy metabolism mainly based on oxidative phosphorylation (Oxphox) [4,7]. This metabolic plasticity of *T*. *cruzi* allows the parasite to survive and colonize different environments, and to establish host infection [8,9].

The vector ingests huge amounts of blood in each meal and is estimated to release almost 10 mM of heme in different forms in the anterior midgut containing the *T*. *cruzi* epimastigote [10]. Heme is an essential porphyrin compound involved in several important biological processes in aerobic organisms. Besides, heme can act as a signaling molecule by directly binding to several cellular factors or realizing products of its metabolism [11,12]. Most trypanosomatids are heme auxotrophs since the parasites partially or completely lack the route for heme synthesis [13,14]. They rely on heme uptake from the host or vector environments [15,16]. The pro-oxidant nature of heme stimulates *T*. *cruzi* epimastigotes to intensely proliferate *in vitro*, resulting in the regulation of processes important to proceed its life cycle, such as differentiation and proliferation of the parasite stage inside the vector [17,18].

The omics era has greatly raised our knowledge of biological molecules. It provides a deeper understanding of organism biology and leads the quest to identify molecules that may be used as targets for therapeutic strategies [19]. Deep transcriptome sequencing characterizing changes in the gene expression of *T*. *cruzi* epimastigotes show that energy metabolism genes associated with glycolysis and aerobic fermentation inside glycosomes are upregulated by heme [20]. Metabolomics is the central omics in the flux of information [21] and has been successfully applied to several biological models [22–26]. Therefore, different biochemical techniques combined with nuclear magnetic resonance (NMR)-based metabolomics were used to better understand glucose metabolism in *T*. *cruzi* epimastigotes cultivated with heme *in vitro*.

## Results

### The proliferation of epimastigotes in the presence of glycolysis inhibitor

**Fig 1:**
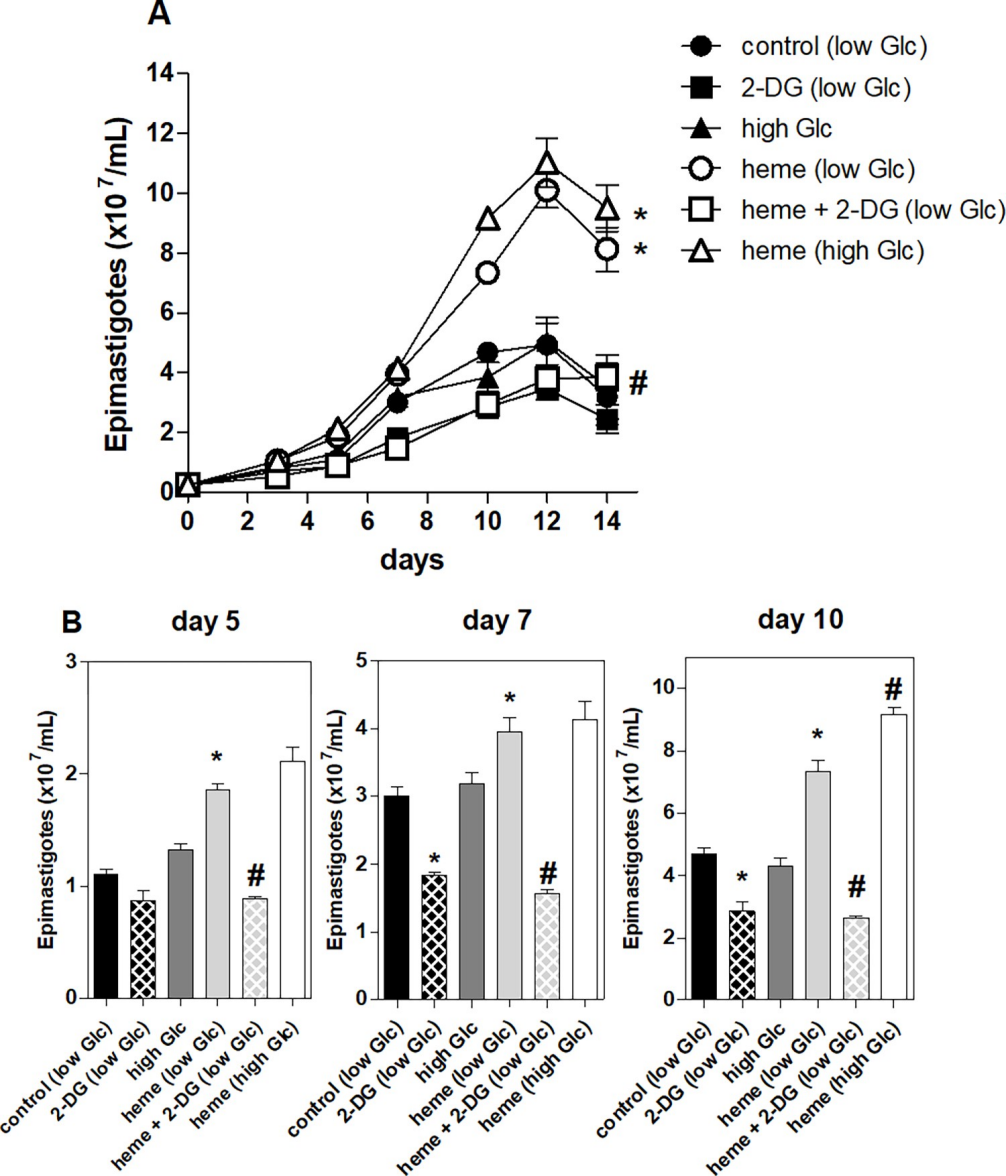
Effect of glycolysis inhibition and supplementation on heme induced epimastigote proliferation. **(A)** *T*. *cruzi* epimastigotes were maintained in brain heart infusion (BHI) media supplemented with 10% fetal bovine serum (FBS) and 30 μM heme at 28°C. The epimastigotes were incubated in the absence or in the presence of 30 μM heme, 20 mM 2-deoxy-glucose (2-DG) in low (6.6 mM) or high (12 mM) D-glucose (Glc) medium for 14 days. **(B)** The proliferation of the parasites was quantified after 5, 7, or 10 days. The data show the mean ± standard error of three independent experiments performed in duplicate. * p <0.05 compared to control group; # p<0.05 compared to 30 μM heme by Tukey’s test.

Heme supplementation induces a significant increase in the expression of glycosomal genes related to glycolysis and aerobic fermentation in *T*. *cruzi* epimastigotes [20] and greatly stimulates the proliferation of *T*. *cruzi* epimastigote forms [17]. This study evaluated parasite growth in the presence of a competitive glycolysis inhibitor [2-deoxy-glucose (2-DG)], in low or high glucose medium. The Brain heart infusion (BHI) medium supplemented with 10% FBS has 6.6 mM of glucose (low glucose medium) **(S1 Table)**. When supplemented with 5.5 mM glucose, the final concentration is ~12 mM (high glucose medium). **Fig 1** shows that 2-DG reduced over 50% of heme-induced proliferation over the days of culture. This effect is evident on days, 5, 7, 10 of culture (**Fig 1)**. 2-DG decreased approximately 40% of epimastigote proliferation after seven days in culture in control parasites grown in low glucose (**Fig 1A**). Parasite proliferation increased after treatment with heme in high glucose medium on day 10 compared with epimastigotes cultured with heme in low glucose However, the addition of 5.5 mM glucose did not affect the proliferation of parasites grown in medium without heme (**Fig 1**). This indicated that epimastigotes depend on glucose metabolism for high heme-induced proliferation.

### Glucose consumption by *T*. *cruzi* epimastigotes in response to heme

**Fig 2:**
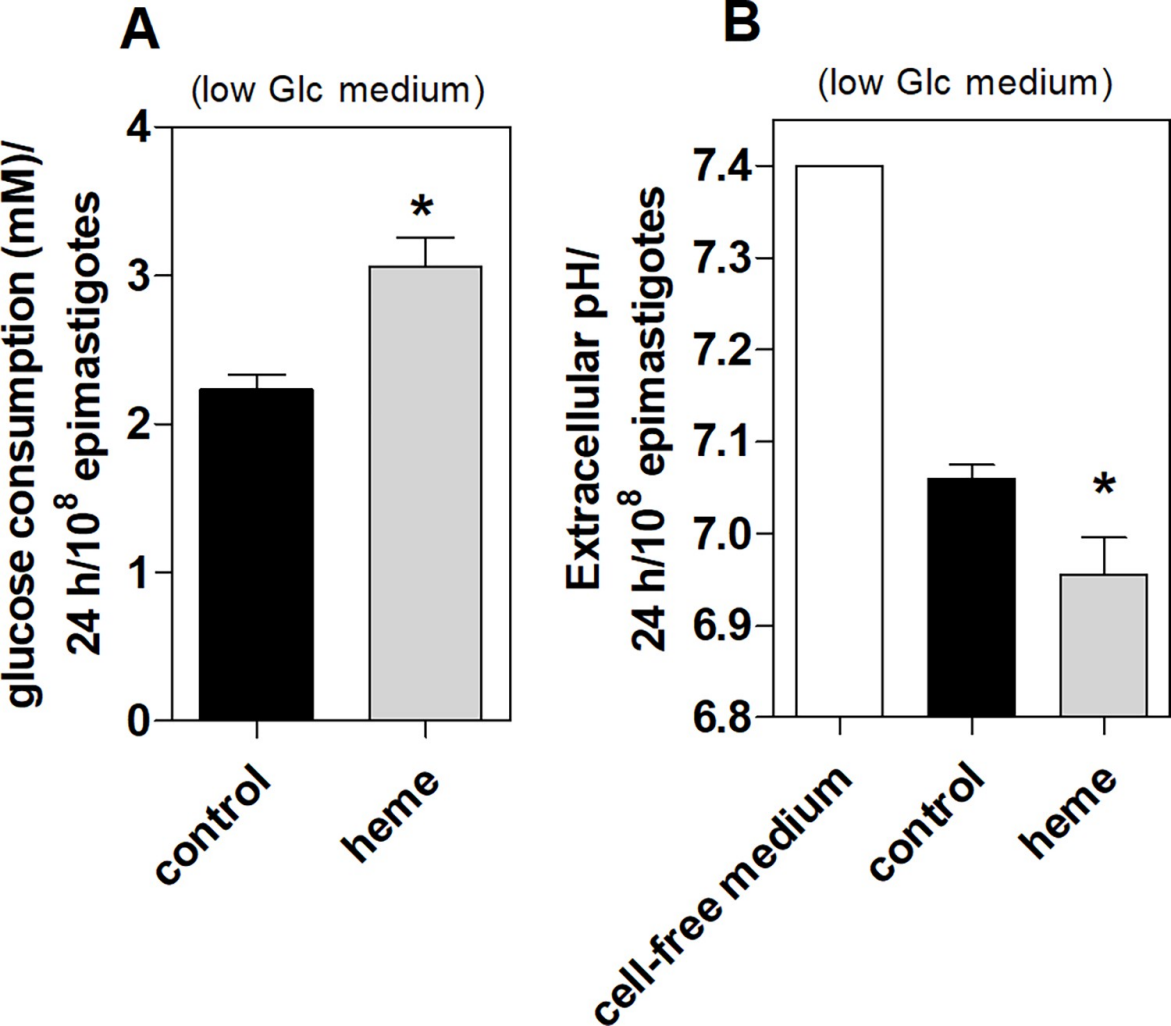
Glucose (Glc) consumption and extracellular pH of epimastigotes treated with heme for 24 h. *T*. *cruzi* epimastigotes were incubated in low Glc (6.6 mM) medium with or without 30 μM heme for 24 h. Afterwards, the culture supernatants were collected and **(A)** Glc concentration was quantified using the Glucose Liquiform kit (Labtest) and **(B)** pH was measured using the pH meter. The parasites were counted in a Neubauer chamber. The data are the mean ± standard error of four independent experiments. *p <0.05 compared to the control group analyzed by Student’s t-test.

The glucose consumption and extracellular pH were evaluated in culture medium containing 6.6 mM glucose. There was a 36% increase in epimastigote glucose consumption compared to control parasites after 24 h incubation of parasites with heme (**Fig 2A**) and was observed a significant decrease in the supernatant pH of these parasites (**Fig 2B**).

**Fig 3:**
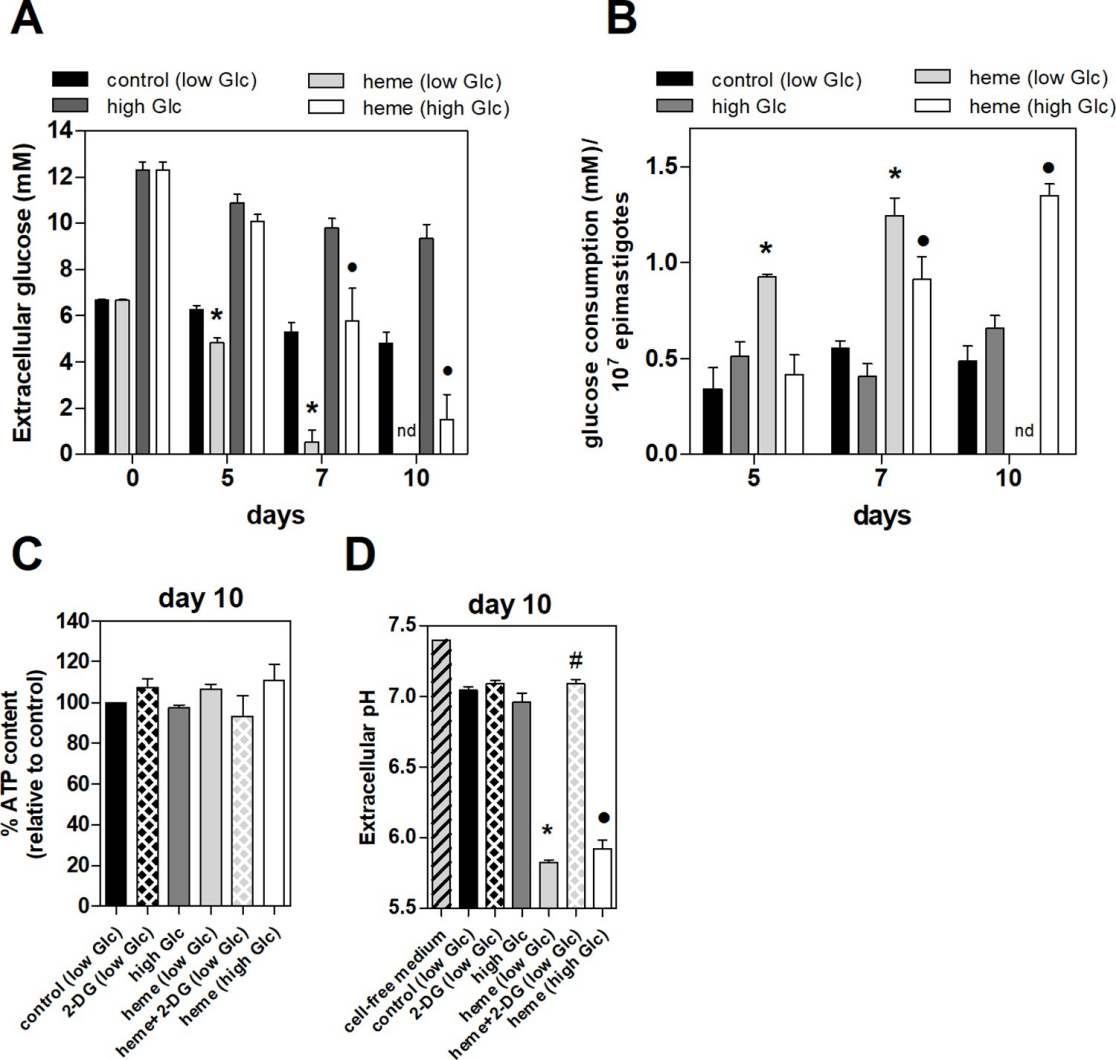
Kinetics of glucose (Glc) consumption and extracellular pH of epimastigotes treated with heme. *T*. *cruzi* epimastigotes were incubated with or without 30 μM heme in low (6.6 mM) or high (12 mM) Glc medium at 28°C for 5, 7, or 10 days. **(A)** Glc concentration was measured from parasite supernatants at each day of culture. **(B)** Glc consumption was obtained by subtracting Glc concentration found in epimastigotes supernatants from day 0 culture medium and normalized by the cell number. **(C)** Percentage of intracellular adenosine triphosphate (ATP) levels of epimastigotes cultured with or without 30 μM heme, 20 mM 2-deoxy-glucose (2-DG) in low (6.6 mM) or high (12 mM) Glc medium for 10 days and **(D)** extracellular pH of these parasites. The results are the mean ± standard error of at least three independent experiments. *p <0.05 compared to control group; #p<0.05 compared to 30 μM heme group; • p<0.05 compared to high Glc group; analyzed by Tukey’s test or Student’s t-test. nd: not detected.

Therefore, the glucose concentration and the pH in the supernatant was evaluated after 5, 7, or 10 days of epimastigote proliferation. Epimastigotes cultivated without heme (control) had a constant glucose consumption rate on day 10; they consumed up to 22% of the total glucose in the medium. Epimastigotes maintained with 30 μM heme for 7 days consumed 92% of the total glucose medium in low glucose medium. No glucose was detected in the supernatants of these parasites on day 10. This indicated that most glucose in the medium was consumed before day 10 (**Fig 3A**). Heme-cultured epimastigotes consumed 88% of the total glucose on day 10 using 12 mM glucose at the start (**Fig 3A**). **Fig 3B** shows the glucose consumption normalized by parasite number at each day of growth. Heme-cultured epimastigotes increased glucose uptake by 2.7-fold and 2.2-fold on day 5 and 7, respectively. The lack of glucose consumption on day 10 suggested that heme-treated parasites uptake glucose until its depletion in the medium; this was not observed in control parasites (**Fig 3B**). Since we observed the total glucose depletion on day 10 **(Fig 3B)**, the intracellular adenosine triphosphate (ATP) levels of epimastigotes were measured in the presence of heme, 2-DG in low and high glucose medium at this culture time. 2-DG impaired heme-induced epimastigote proliferation (**Fig 1A and 1B**); however, ATP levels were not significantly different among parasites groups, even with glucose supplementation in the medium **(Fig 3C)**. The pH of heme-cultured epimastigote supernatants significantly decreased on day 10 compared to control or high glucose supernatants. Interestingly, this extracellular acidification of heme-cultured parasites was not observed in the presence of 2-DG, which had a similar supernatant pH to that of the control culture medium **(Fig 3D)**. This supports the idea that heme-cultured epimastigotes increase aerobic fermentation.

### The effect of exogenous D-glucose on epimastigote mitochondrial physiology

**Fig 4:**
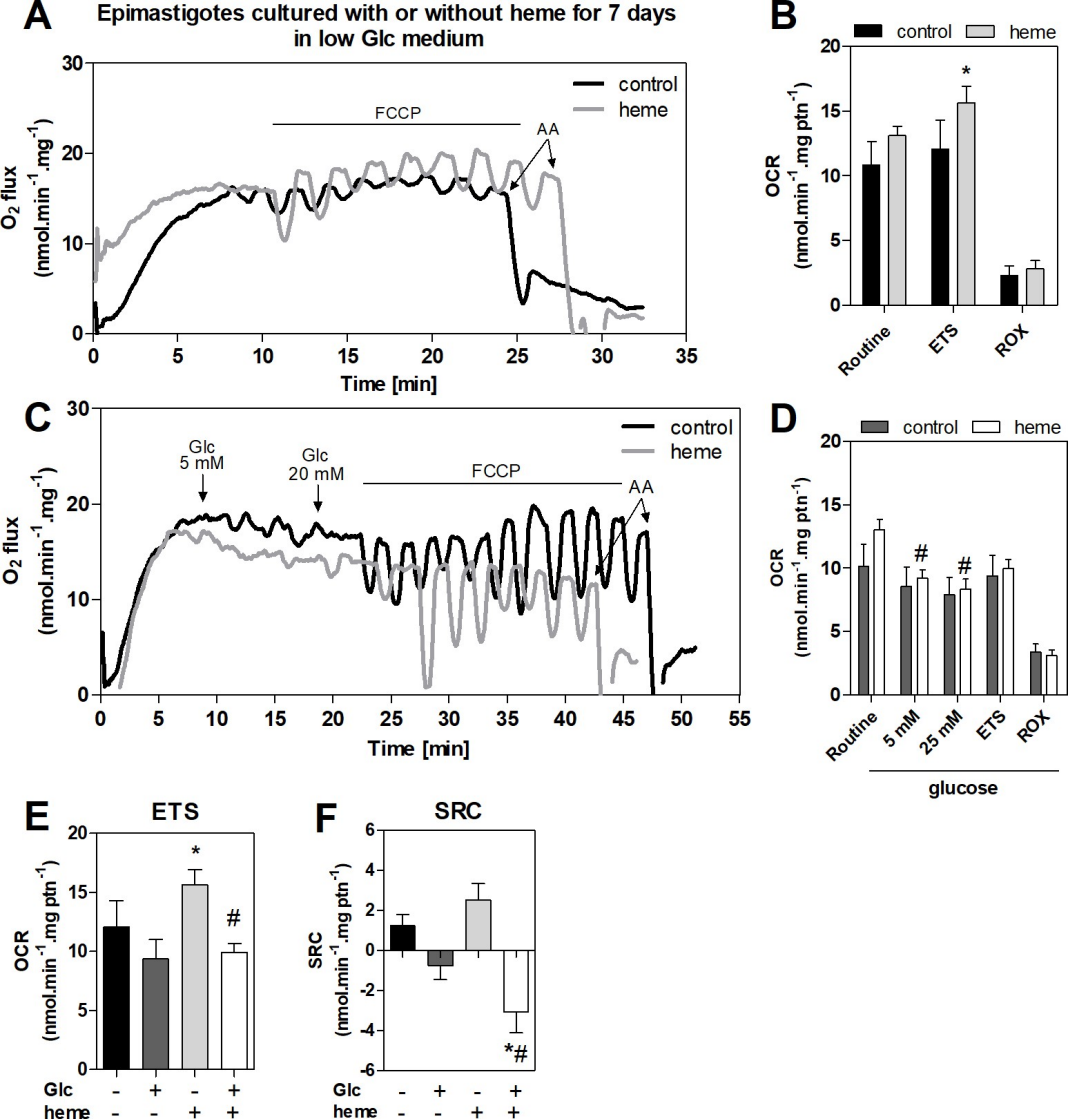
Oxygen consumption of heme-cultured epimastigotes in the presence of exogenous glucose (Glc). *T*. *cruzi* epimastigotes were grown in low Glc (6.6 mM) medium with or without 30 μM heme at 28°C for 7 days and the oxygen consumption rate (OCR) of parasites was evaluated by high-resolution respirometry. **(A)** Representative OCR traces of epimastigotes indicating the titration of carbonyl cyanide p-(trifluoromethoxy) phenylhydrazone (FCCP) and 2 μg/mL antimycin A (AA). **(B)** Routine OCR; maximal oxygen consumption (electron transport system related OCR; ETS) after FCCP titration; and residual respiration (ROX) obtained after AA. **(C)** Representative OCR traces of epimastigotes with Glc addition. Increasing concentrations of Glc (5–20 mM) were followed by up to 300 nM of FCCP and 2 μg/mL AA where indicated. **(D)** Routine OCR and OCR after 5 mM and 20 mM Glc addition. The ETS OCR and ROX after Glc exposure were also calculated. *p<0.05 of each parameter compared to control group analyzed by the unpaired Student’s t-test. #p<0.05 compared to routine OCR of heme parasites analyzed by analysis of variance (ANOVA) with post hoc Dunnett’s. **(E)** ETS OCR and **(F)** spare respiratory capacity (SRC) of epimastigotes submitted **(+)** or not **(-)** to Glc. All data are the mean ± standard error of at least six independent experiments. *p <0.05 compared to control OCR (- Glc); •p <0.05 compared to control OCR after Glc addition (+ Glc); #p <0.05 compared to heme OCR (- Glc); analyzed by Student’s t-test.

High resolution respirometry was performed in heme treated epimastigotes challenged with increasing concentrations of glucose, or without glucose (control) to assess the effect of glucose supplementation on epimastigote mitochondrial physiology. The routine oxygen consumption rate (OCR) was similar in heme and control parasites between 0–10 days (**Figs 4A, 4B and S1A**). The classical Crabtree effect was observed after glucose addition (the glucose-mediated inhibition of mitochondrial respiration) [27] with the reduction of epimastigote O_2_ consumption. Heme treated parasites presented greater respiration inhibition at all time points compared to control cells (**Figs 4C, 4D and S1B**).

The maximum OCR (electron transfer system, ETS) was stimulated by the addition of carbonyl cyanide p-(trifluoromethoxy) phenylhydrazone (FCCP) and heme-treated parasites presented an increment of 30% ETS OCR compared to control epimastigotes (**Fig 4A and 4B**). This increment in the ETS was impaired by glucose addition **(Fig 4D)**, with a 36.5% reduction in the maximum oxygen consumption (**Fig 4E**).

The residual oxygen consumption (ROX) is the mitochondrial non-electron transport system respiration that is achieved after the addition of Antimycin A (AA). It was not altered in epimastigotes cultured with heme compared to control parasites in any assay **(Fig 4B and 4D)**.

The spare respiratory capacity (SRC) reflects an estimative of the cell’s ability to cope with large increases in energy demand. It only decreased in heme treated parasites challenged with glucose. This suggested these parasites (but not control epimastigotes) require ATP produced by the ETS in the absence of glucose to maintain plasticity in responding to energetic stress (**Fig 4F**).

### NMR-based metabolomics of intracellular and extracellular metabolites of epimastigotes maintained with heme and D-glucose

**Fig 5:**
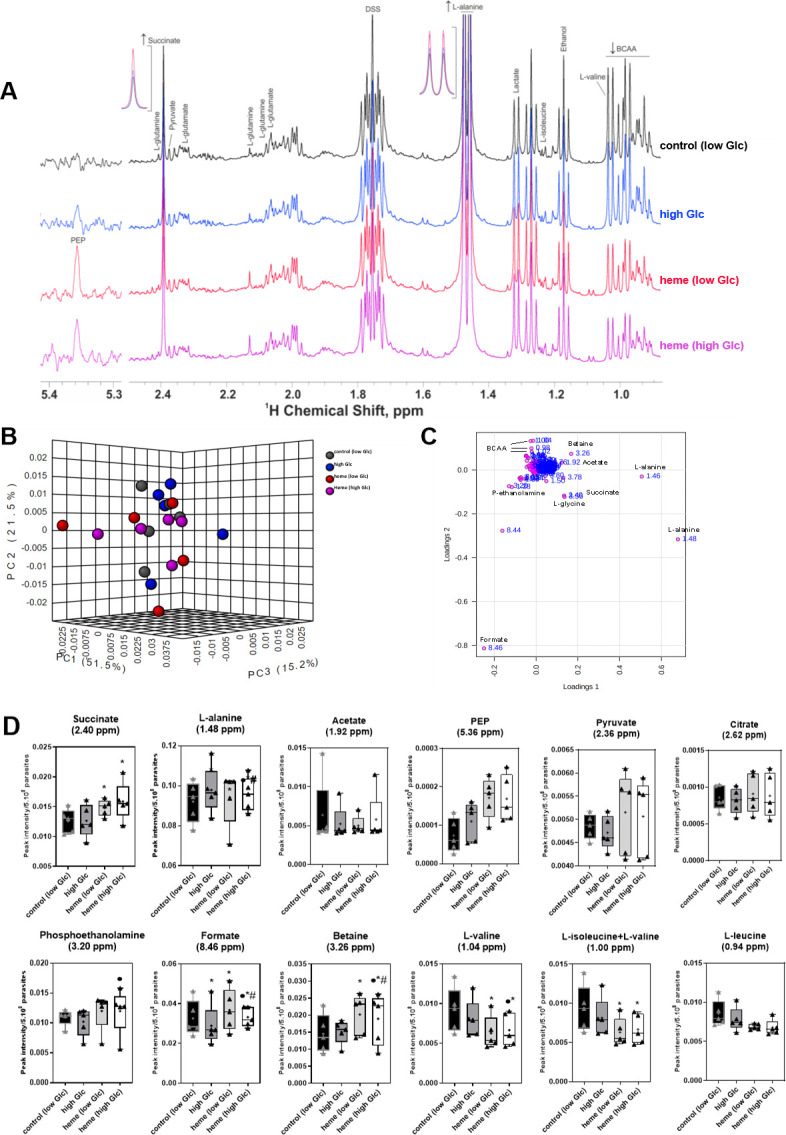
NMR-based metabolomics of *T*. *cruzi* epimastigotes cultured for 7 days with heme and glucose (Glc). *T*. *cruzi* epimastigotes were cultured in low (6.6 mM) or high (12 mM) Glc medium with or without 30 μM heme at 28°C for 7 days. Polar metabolites were extracted from 5x10^8^ parasites, and the molecular composition of each sample was analyzed by NMR. **(A)** Representative ^1^H-NMR spectra of polar metabolites of parasites extracts. **(B)** Principal component analysis score plot and **(C)** loading plot of epimastigotes extracts from five independent experiments **(S4 Table)**. **(D)** Box plots showing peak intensity of each metabolite found in the spectrum with its ^1^H chemical shift (ppm). Data are the mean ± standard error. *q<0.05 compared to control parasite; #q<0.05 compared to heme parasite; ●q<0.05 compared to high Glc parasite; analyzed by the two-way analysis of variance with a false discovery rate approach (q value<0.05) calculated by the Bejamini, Krieger, and Yekutiele method **(S5 Table).**

^1^H NMR-based metabolomic analysis were carried out to investigate the intracellular and extracellular metabolome of *T*. *cruzi* epimastigotes cultured with heme for 7 days. **Fig 5** shows analysis of several proton signals from polar metabolites of epimastigotes extracts, assigned and identified by the [^1^H-^1^H] TOCSY (Total Correlation Spectroscopy) experiment (**S2 and S3 Tables**). Representative ^1^H-NMR spectrum of parasites cultivated with or without heme in low or high glucose medium are represented in **Fig 5A**. A representative ^1^H-NMR spectrum of aromatic metabolites of these parasite samples is shown in **S2A Fig**.

The principal component analysis (PCA) score plot of data from parasite extract samples demonstrates similarity between heme-cultured parasites and parasites cultured without heme (**Figs 5B and S2B**). This suggests that the most of metabolites remain relatively unchanged among the conditions in the multivariate analysis. The PCA loading plot, otherwise, pinpointed specific metabolites that exhibited differences between the heme-containing groups and the control, and these differences were found to be statistically significant in the univariate analysis **(Fig 5C and S4 Table**).

Heme-cultured epimastigotes exhibit an increase in succinate (1.18-fold), L-alanine (1.06-fold), formate (1.13-fold) and betaine (1.39-fold) compared to control parasites (**S5 Table**). The decreased metabolites in heme-cultured parasites included L-valine (0.68-fold) and L-isoleucine + L-valine (0.67-fold) (**Fig 5D**). L-leucine had similar levels between heme and control parasites grown in low glucose conditions. Interestingly, parasites grown in high glucose medium (12 mM) without heme for 7 days enhanced intracellular L-alanine (1.06-fold) and decreased formate (0.89-fold). Heme-cultured parasites containing 12 mM glucose medium increased intracellular levels of L-alanine by 1.02-fold and decreased betaine (0.94-fold) and formate (0.90-fold) compared to parasites cultured with heme. Related to phospholipids, phosphoethanolamine increased (1.17-fold) in parasites cultured with heme in high glucose medium (12 mM) compared to parasites grown without heme **(Fig 5D)**.

**Fig 6:**
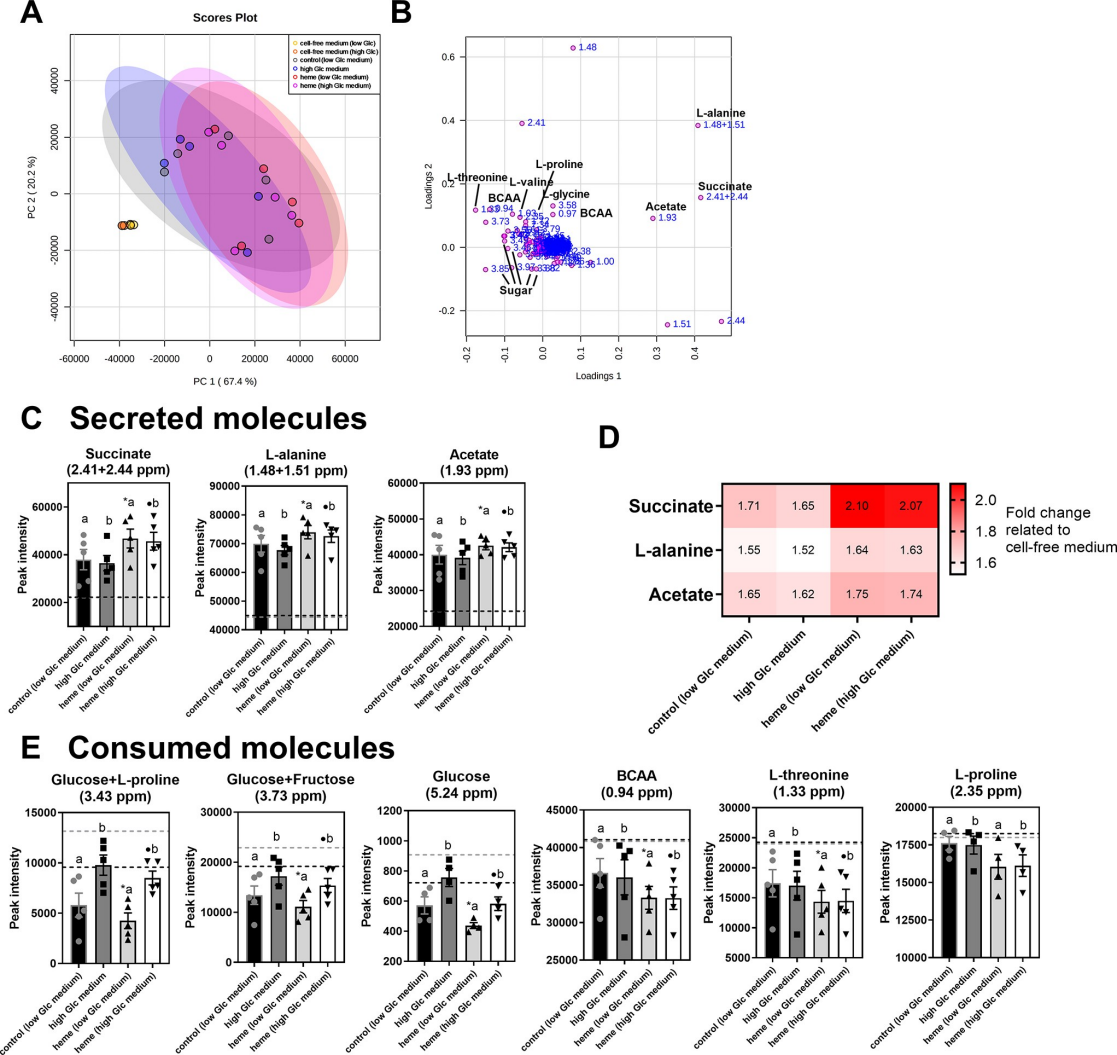
NMR-based metabolomics of the culture supernatants of epimastigotes grown for 7 days. (**A)** Principal component analysis score plot and **(B)** loading plot of four independent experiments **(S6 Table). (C)** Box plots showing the peak intensity of secreted molecules in parasite supernatants after 7 days of culture. **(D)** Heatmap representing the fold change of secreted molecules. The fold change was calculated by metabolite intensity measured in parasite supernatants relative to metabolite intensity found in cell-free medium. **(E)** Box plots show the peak intensity of metabolites which exhibited low levels compared to cell-free medium (consumed molecules). The results show mean ± SE. *Dashed line* is the peak intensity of the molecule found in low (6.6 mM) Glc cell-free medium (black line) and high (12 mM) Glc cell-free medium (gray line). *a* q<0.05 related to low Glc cell-free medium; *b* q<0.05 related to high Glc cell-free medium **(S7 Table)**; *q<0.05 compared to control parasites medium; ●q<0.05 compared to high Glc medium **(S8 Table)**; analyzed by the Two-way ANOVA with FDR approach calculated by the Bejamini, Krieger and Yekutiele method. q-value<0.5 with fold change>1 = secreted molecules to the medium; q-value<0.05 with fold change<1 = consumed molecules from the medium.

Extracellular metabolites of parasites cultivated in these four conditions were also analyzed. Initially, a ^1^H-^13^C HSQC (Heteronuclear Single Quantum Coherence) spectra was acquired to identify the molecules present in cell-free medium (BHI supplemented with 10% FBS and 6.6 mM glucose) (**S1 Table**). The molecules found in epimastigote-cultured medium were compared to molecules present in cell free medium using 2D NMR (**S4 Fig and S7** and **S8 Tables**). Multivariate analysis showed that molecules of the culture medium without cells were remarkably altered by parasites (**Fig 6A and 6B and S6 Table**). Regarding the secreted molecules, heme group presented a rise in succinate (2.10-fold), acetate (1.75-fold), and L-alanine (1.64-fold) compared to the cell-free medium (**Fig 6C and 6D**). Heme-cultured epimastigote secreted higher levels of succinate (1.71-fold), acetate (1.64-fold), and L-alanine (1.53-fold) in culture medium compared to the supernatant of control parasites (**Fig 6C and 6D**). Sugars were the most consumed molecules by all parasites **(Fig 6E and Table 1**). The peaks specifically relative to glucose (but not other hexoses or disaccharides) were determined after comparing the cell-free medium of low and high glucose conditions **S7 Table).** Heme-cultured epimastigotes had high consumption of glucose and other sugars compared with control parasites and reached a maximum difference of ~17% between the supernatants **(Tables 1 and S8 and Figs 6E and S3)**. The most consumed amino acids by heme-cultured parasites after 7 days were L-threonine, branched-chain amino acids, and L-proline (**Tables 1 and S8 and Fig 6E**). Collectively, the supernatant of heme-cultured parasites was poorer in nutrients and higher in organic acids secreted by epimastigotes after 7 days (**S7 Table)**. Together, these results suggested that glycolysis and succinic acid fermentation activity is enhanced in *T*. *cruzi* epimastigotes in response to heme treatment.

**Table 1:** The most consumed molecules relative to cell-free medium.

Molecules	^1^H Chemical Shift	low Glc medium		high Glc medium	
		control	heme	high Glc	heme
**glucose +L-proline**	3.43 ppm	0.61	0.44	0.74	0.65
**glucose +fructose**	3.73 ppm	0.70	0.58	0.75	0.67
**L-threonine**	1.33 ppm	0.72	0.59	0.71	0.60
**glucose**	5.24 ppm	0.79	0.61	0.83	0.64
**BCAA**	0.94 ppm	0.89	0.81	0.89	0.82
**L-proline**	2.35 ppm	0.91	0.83	0.91	0.85

## Discussion

There is an evolutionary pressure for unicellular organisms to multiply as quickly as possible when nutrients are available in the environment. Glucose can be used to generate biomass and to produce ATP during growth [28]. Here, the increased glucose intake by heme-treated parasites was not converted into large amounts of intracellular or extracellular pools of fermentation-related products. This indicated that this sugar may be shunted for anabolic pathways to sustain epimastigote replication. This idea is supported by the observation that only glucose supplementation in the medium enabled the proliferation of heme-cultured epimastigotes, while the depletion of glucose from the supernatant delayed their growth. Thus, there is a metabolic reprogramming in these parasites that probably allows switching its energy metabolism to use amino acids as an energy source [7]. This shift supports ATP production in these parasites even with inhibited glycolysis, although the growth is limited.

These protozoans partially catabolize the glucose and secrete reduced compounds such as succinate and L-alanine to the environment under aerobic conditions, instead of complete oxidation to CO_2_. Succinate formation occurs through the glycosomal ‘succinic branch’ formed by phosphoenolpyruvate carboxykinase (PEPCK)—malate dehydrogenase (MDH)—fumarate reductase (FRD) from phosphoenolpyruvate (PEP) produced in the cytosol, whereas L-alanine and acetate are produced from pyruvate [4,29]. These three enzymes of the succinic branch (and enolase) are upregulated in *T*. *cruzi* in response to heme treatment [20]. This study showed that glucose consumption was enhanced, together with intracellular levels of succinate (but not pyruvate and citrate). This highlights that heme-cultured epimastigotes present an increase in the activity of succinic acid fermentation. Mitochondrial respiration does not follow the increase in glucose intake by heme-cultured parasites. This provides supporting evidence that Glc metabolism is not related to the mitochondrial route. The preference of the glycolytic pathway is well-known in yeasts, some tumor cells, and other parasitic organisms. These cells have a functional electron transport system in their mitochondria; however, they ignore the O_2_ and synthesize ATP through substrate-level phosphorylation in glycolysis to secrete partially oxidized products, like succinate, acetate, L-alanine, pyruvate, ethanol, and/or L-lactate [30]. Thus, there is strong indication that glycolysis fulfills its energy requirement for growth in heme-cultured epimastigotes while succinic acid fermentation sustains redox and ATP/ADP balance in glycosomes.

Heme acts as an important regulator. It induces a metabolic adaptation through the glycolytic phenotype and fermentation was reported in *Aedes aegypti* midgut cells [31]. The same authors observed that heme exposure upregulates the expression of energy metabolism associated genes of glucose catabolism and prompts enhanced lactate production in heme-incubated cells or midguts of heme-fed mosquitoes; this supports other results [20]. This metabolic switch in blood-feeding organisms is associated with the oxidant nature of heme, which provides a carbon source to the pentose phosphate pathway to help prevent oxidative burst in the mosquito’s intestine [31]. The metabolic re-wiring towards aerobic glucose fermentation in the oxygen-rich environment was also observed in blood-feeding organisms, including *Schistosoma*, *Angiostrongylus*, and *Plasmodium* [32].

Heme intake by *T*. *cruzi* is essential for synthesizing heme proteins like cytochromes that are part of mitochondrial complexes [11,14]. Meanwhile, the lack of heme supplementation of control parasite medium could affect the abundance of its cytochromes. This may be reflected through minor O_2_ consumption. Here, parasites cultured with heme for 7 days presented an increase in O_2_ consumption related to the ETS involving mitochondrial metabolism, unlike phenomenon previously demonstrated by Nogueira et al. [33], where cells were incubated for 30 minutes. Little is known regarding the flexibility level of these parasites in response to changes in their immediate environment. This work evidenced that heme-cultured parasites suddenly react to glucose presence. The addition of glucose as the sole substrate triggered an inhibition of the OCR related to maximal electron transfer and capacity to produce ATP by oxidative respiration in *T*. *cruzi* cultured with heme. The glucose-induced repression in mitochondrial respiration is suggestive of the Crabtree-*like* effect phenomenon referring to cell adaptation-based on aerobic glycolysis, away from Oxphox when in the presence of glucose [27]. This metabolic adaptation is widely known for numerous cell types, particularly in proliferating cells, like tumor cells, bacteria, and yeast. This reinforces evidence showing that glycolysis supports the energy requirements of an epimastigote induced by heme.

Trypanosomatids prefer carbohydrates for energy; however, they can proliferate using amino acids, fatty acids, and glycerol [5,6,34,35]. L-threonine is the most consumed carbon source in *T*. *brucei* grown in glucose-depleted medium at the insect stage. The degradation of L-threonine in *T*. *brucei* and L-leucine (a branched-chain amino acids) and acetate in *T*. *cruzi* produces acetate that the parasites use for sterol synthesis, and it is required for their membranes [36,37,38]. Thus, the metabolism of these specific amino acids could sustain lipid synthesis required for proliferation.

The mechanism by which epimastigotes proliferate in the presence of large amounts of heme is here shown to be dependent on glucose, resulting in metabolic adaptations exclusive to the parasite to obtain energy. These point to new therapeutic targets, specifically those involved in the successful infection of the hematophagous insect by *Trypanosoma cruzi*. The interruption the cell cycle of the Chagas disease agent can impair the vectorial transmission, the main mode of spreading in endemic countries.

**Fig 7:**
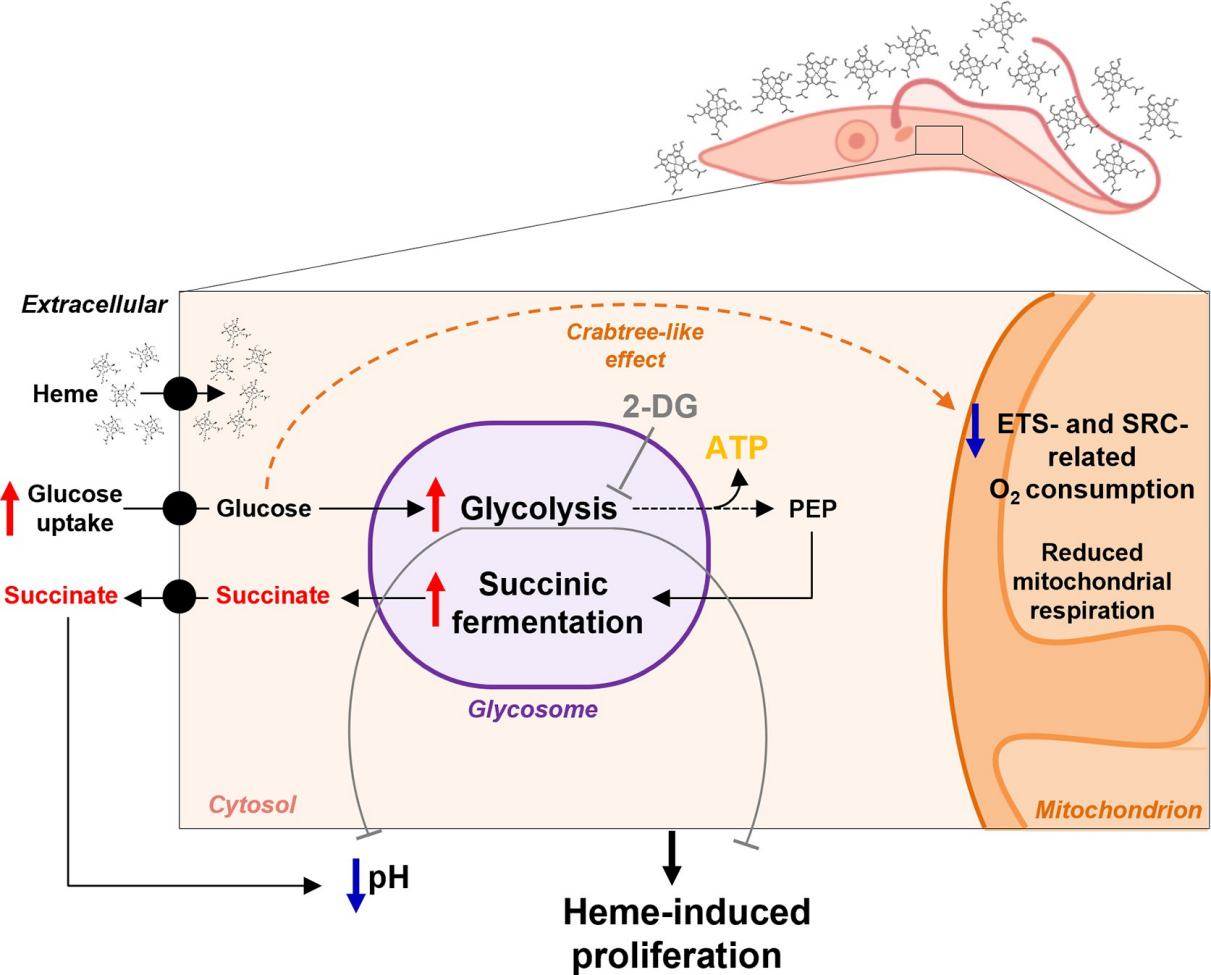
Proposed scheme of glucose metabolism of *T*. *cruzi* epimastigotes stimulated by heme. *T*. *cruzi* epimastigotes increase proliferation and upregulation of the expression of glycosomal genes related to glycolysis and aerobic fermentation in response to heme [17,20]. Here, the aerobic fermentation of glucose overcomes mitochondrial metabolism in epimastigotes grown with heme. *T*. *cruzi* epimastigotes have enhanced glucose consumption after heme treatment, and mainly produce and secrete succinate as the final product of succinic acid fermentation. High succinate levels decreased the extracellular pH, leading to an acidification of the supernatant. However, this acidification and the great proliferation induced by heme was impaired when glycolysis was inhibited by 2-deoxy-glucose (2-DG) (*gray lines*). This indicates that succinate and other fermentation-related molecules are products of glycolysis, and this metabolic pathway is essential for the increment of parasite growth. In heme-cultured parasite an increase in glucose intake does not result in increased mitochondrial respiration. Mitochondrial O_2_ consumption related to electron transport system (ETS) is immediately reduced after glucose addition to present a Crabtree-*like* effect allowing organisms to maintain its energy metabolism towards aerobic glucose fermentation.

A schematic representation of the proposal metabolic adaptation influenced by heme of *T*. *cruzi* epimastigotes is presented in **Fig 7**. Collectively, these data substantiate the idea that heme signaling modulates the energy metabolism of *T*. *cruzi* epimastigotes and promotes a metabolic adaptation towards aerobic fermentation of glucose, while negatively regulating oxidative metabolism in the presence of sugar to sustain higher proliferation.

## Conclusion

It is known that hemin supplementation in the culture medium increases the growth of the epimastigote forms of *T*. *cruzi*. This paper demonstrates that heme-induced growth is dependent on glucose, with fermentative metabolism predominating over mitochondrial respiration. This demonstrates the ability of the parasite to adapt to variations throughout its life cycle.

## Materials and methods

### Parasites

*T*. *cruzi* epimastigotes (CL Brener) were provided by the Trypanosomatid Collection of the Oswaldo Cruz Institute, Fiocruz, Brazil. They were grown at 28°C for 7 days in BHI medium (BD Bacto, USA), supplemented with 10% FBS (Vitrocell, Campinas, Brazil) in the presence of 30 μM heme (Frontier Scientific, Utah, USA).

### Heme

20 mM heme (Fe–protoporphyrin IX) stock solution was freshly prepared by dissolving in 0.1 N NaOH, followed by buffering using PBS (100 mM sodium phosphate buffer and 150 mM NaCl at pH 7.4). The stock solution was immediately diluted before use to 5 mM PBS.

### *In vitro* proliferation assay

Epimastigotes (2.5 × 10^6^ parasites/mL) were grown at 28°C for 14 days in BHI medium supplemented with 10% FBS (6.6 mM glucose) in the presence or absence of 30 μM heme, 20 mM 2-DG or—supplemented with more 5.5 mM glucose(Sigma-Aldrich, St. Louis, MO) reaching a total glucose ~12 mM. Parasite growth was monitored by cell counting in a Neubauer chamber. Three independent experiments were performed in duplicate.

### D-glucose consumption and extracellular pH

Cells were inoculated at 2.5 x 10^6^ cells/ml and grown in 6.6 mM (low) or 12 mM (high) glucose medium in the presence or absence of 30 μM heme to determine the rate of glucose consumption. Aliquots of each growth medium were collected after 5, 7, or 10 days after incubation at 28°C. Alternatively, 1 × 10^8^ epimastigotes were treated with or not 30 μM heme for 24h. The quantity of glucose in the supernatants was determined using the Glucose Liquiform kit (Labtest, Minas Gerais, Brazil). The glucose consumption was determined by calculating the difference between the glucose concentration in cell-free medium and in the supernatant after the parasite culture. The values were normalized by cell numbers. The pH of the supernatants was evaluated using a pH 300 M Analyzer pH meter (São Paulo, Brazil).

### ATP quantification

Intracellular ATP levels were measured by the CellTiter-Glo luminescent cell viability assay (Promega, Wisconsin, USA) where the signal is proportional to the ATP concentration [33] with minor modifications. Briefly, epimastigotes were grown in 6.6 mM or 12 mM glucose medium in the presence or absence of 30 μM heme or 20 mM 2-DG for 10 days. The parasites were washed in PBS, and the parasite concentration was adjusted to 1 × 10^7^ cells in 200 μL of PBS. Then 50 μL was transferred onto opaque-walled 96-well plates and 50 μL of the CellTiter-Glo reagent was directly added into each well. The plates were incubated in the dark for 10 min and the luminescence was read using an Envision multimode plate reader (Perkin-Elmer, Massachusetts, USA). ATP concentrations were calculated from the ATP standard curve.

### Oxygen consumption rates

Epimastigotes (2.5 × 10^6^ parasites/mL) were maintained with or without 30 μM heme at 28°C for 5, 7, and 10 days. O_2_ consumption rates were evaluated by high-resolution respirometry (Oxygraph2K; Oroboros Instruments, Innsbruck, Austria) under continuous stirring. The temperature was maintained at 28°C and intact parasites (5 × 10^7^ parasites/chamber) were added in 2 mL of Krebs-Henseleit buffer (KHB; 111 mM NaCl, 4.7 mM KCl, 1.25 mM CaCl_2_, 2 mM MgSO_4_, 1.2 mM Na_2_HPO_4_, pH 7.2) [39]. Increasing concentrations of glucose were added (5.5–20 mM) for substrate response assays and the Crabtree effect. The glucose-mediated inhibition of mitochondrial respiration (ΔOCR) was calculated by the difference in the respiration after glucose addition and routine respiration. The noncoupled state of maximum respiration was induced by the addition of up to 300 nM of FCCP (Sigma-Aldrich, St. Louis, MO, USA) to allow for the maximal capacity of the ETS to be measured. Respiration was inhibited by the addition of 2 μg/mL AA, a complex III inhibitor (Sigma-Aldrich, St. Louis, MO, USA) to determine ROX. The physiological OCR (Routine) and the ETS maximal capacity data were calculated by subtracting the ROX consumption values from the initial OCR and after the addition of FCCP, respectively. Finally, the SRC was calculated by subtracting ETS from routine respiration [40]. Protein concentration was determined by the Lowry method, using bovine serum albumin as standard [41]

### NMR-based metabolomics

Epimastigotes (2.5 × 10^6^ parasites/mL) were grown in 6.6 mM (low) or 12 mM (high) glucose medium with or without 30 μM heme at 28°C for 7 days. The culture supernatants were collected and frozen for NMR analysis. 5 × 10^8^ epimastigotes were washed twice in ice-cold PBS and centrifuged at 4°C. The cell pellets were submitted to the biphasic extraction method consisting of methanol:chloroform:water (1:1:1 v/v/v). The polar phase was dried using a SpeedVac and stored at -80°C. Parasite extracts were resuspended in 50 mM sodium phosphate buffer (pH 7.4), with 1 mM 4,4-dimethyl-4-silapentane-1-sulfonic (DSS) acid as the internal standard, and 10% D_2_O. Only 10% D_2_O was added for culture supernatants. Nuclear magnetic resonance spectroscopy was conducted on a Bruker Avance III instrument operating at the ^1^H frequency of 500.13 MHz at 298 K. Parasite extract samples were analyzed by acquiring 1D ^1^H spectra using a ^1^H-zgesgp pulse sequence with 512 scans, size of FID of 64K, SW of 20 ppm, acquisition time of 3.27 seconds, transmitter frequency offset of 4.7 ppm, RG of 203. Culture supernatant samples were analyzed using a ^1^H-zgesgp pulse sequence with a Carr-Purcell-Meiboom-Gill filter to suppress undesired signals from high molecular weight molecules and 512 scans were acquired. Furthermore, 2D [^1^H-^13^C] HSQC spectra and 2D [^1^H-^1^H] TOCSY were acquired to assign metabolites present in cell-free culture medium (**S1 Table**) and parasite extract samples (**S2 and S3 Tables**), respectively. All 1D spectra were processed with 128K points, phase, baseline corrected and calibrated by DSS or, and aligned in TopSpin 4.0.7 (Bruker-Biospin). HMDB 4.0, BMRB, and COLMARm databases were used for assignments [42–45]. In addition, all spectra were binned with an average SUM method at 0.02 ppm for the extract sample, and at 0.03 ppm for medium samples using MestReNova software. Water, ethanol, and the solvent bucket were deleted for analysis. Extract sample (parasite) data was normalized by the sum of intensities.

### Statistical analysis

Statistical analyses were conducted with GraphPad Prism 5 or Prism 7 software (GraphPad Software, Inc., San Diego, CA). Data are presented as the mean ± standard error (SE), and all experiments were independently performed at least three times. Data were analyzed by one-way ANOVA, and differences among groups were assessed with Tukey’s or Dunnett´s post-tests. An unpaired Student’s t-test was used when necessary. Metabolomic data was analyzed by two-way ANOVA with a false discovery rate (FDR) (Q = 5%) approach calculated by the Bejamini, Krieger, and Yekutiele method. Multivariate analysis was carried out on MetaboAnalyst 5.0 [46]. The level of significance was set at p<0.05.

## Supporting information

S1 Fig Effect of exogenous glucose on epimastigote oxygen consumption rates. *T*. *cruzi* epimastigotes were grown in medium with or without 30 μM heme at 28°C for 5, 7, and 10 days and oxygen consumption rates (OCRs) of epimastigotes (5 × 10^7^ parasites/chamber) were evaluated by high-resolution respirometry. **(A)** Routine OCR, **(B)** Crabtree effect obtained by the difference between respiration after Glc addition (20 mM) and routine OCR (ΔOCR); and **(C)** maximal respiration (electron transport system, ETS) stimulated by increasing concentrations of FCCP. The results show the mean ± standard error of at least four independent experiments. *p<0.05 compared to control parasites analyzed by unpaired Student’s t-test.
(TIF)

S2 Fig Aromatic region of ^1^H-NMR spectrum and Principal component analysis (PCA). **(A)** Representative ^1^H-NMR spectrum of intracellular metabolites in the aromatic region of epimastigotes cultured in four conditions. **(B)** Multivariate analysis of the metabolome of four epimastigote conditions evaluated in the study. 2D-principal component analysis (PCA) score plot of the epimastigote sample extract. The figure was generated using MetaboAnalyst 5.0 software.
(TIF)

S3 Fig Representative ^1^H-NMR spectrum of epimastigote supernatants. Epimastigotes were cultured for 7 days vs. **(A)** cell-free medium with low Glc [BHI supplemented with 10% fetal bovine serum (FBS); 6.6 mM Glc], *yellow line* or **(B)** cell-free medium with high Glc (12 mM Glc), *orange line*.
(TIF)

S1 Table Peak report of the [^1^H-^1^H] TOCSY spectrum. The data is from the heme treated epimastigote extract (generated by COLMAR). The columns show the proton and carbon chemical shift peak values, the peak amplitude values and compound names.
(XLSX)

S2 Table Compound report of the [^1^H-^1^H] TOCSY spectrum. The data is from the heme treated epimastigote extract (generated by COLMAR). The compound names, matching ratio in the spectrum, and its database ID (Source) are indicated. The source column was used to confirm the compound in the 1D spectra.
(XLSX)

S3 Table Peak and compound report of the [^1^H-^13^C] HSQC spectrum. The result is from the cell-free medium sample generated by COLMAR. The first two columns show the proton and carbon chemical shift peak values, respectively. The third column shows the peak amplitude values. The last columns show the compound names matched with the respective ^1^H-^13^C chemical shift.
(XLSX)

S4 Table Principal component analysis (PCA) of parasite extract samples. Multivariate analysis were generated by MetaboAnalyst 5.0.
(XLSX)

S5 Table Two-way analysis of variance (ANOVA) of parasite extract samples. Table presents metabolites identified from NMR spectra, proton chemical shift value (ppm), and fold change analysis between the groups. p value and q values were obtained by two-way ANOVA, with a 5% false discovery rate (FDR) approach using the Benjamini, Krieger, and Yukitieli method. q<0.05 (*Yes*) show the status of the metabolite relative to each sample (2 vs. 1) and was expressed as ↓ (decreased) or ↑ (increased). The data was represented in Fig 5.
(XLSX)

S6 Table Principal component analysis of culture supernatants. Multivariate analyses were generated by MetaboAnalyst 5.0.
(XLSX)

S7 Table Two-way analysis of variance (ANOVA) of culture supernatants. The peak intensity of metabolites identified in cell-free medium in low Glc (6.6 mM) medium in *yellow* or in high Glc (12 mM) medium in *orange* vs. parasites media. ↓ corresponds to decreased metabolite levels relative to cell-free medium (consumption); ↑ corresponds to increased metabolite levels relative to cell-free medium (secretion). All metabolites between parentheses were molecules identified using the Human Metabolome Database (HMDB) and the Biological Magnetic Resonance Bank (BMRB) database. *Metabolites are molecules with two peaks sum. The data are represented in **Fig 6**.
(XLSX)

S8 Table Two-way analysis of variance (ANOVA) of culture supernatants II. Statistical analysis of metabolites intensity between epimastigote supernatants samples. The table has the compound name with its proton chemical shift value, fold change analysis, *p*-value, *q*-value, and status of the metabolite relative to the samples. *Metabolites are molecules with two peaks sum. The data are represented in **Fig 6**.
(XLSX)
