# Supplementary figures and images for: Glucose metabolism sustains heme-induced Trypanosoma cruzi epimastigote growth in vitro
Source: PLoS Negl Trop Dis. 2023 Nov 10;17(11):e0011725. doi: 10.1371/journal.pntd.0011725 (PMC10664871; doi:10.1371/journal.pntd.0011725)

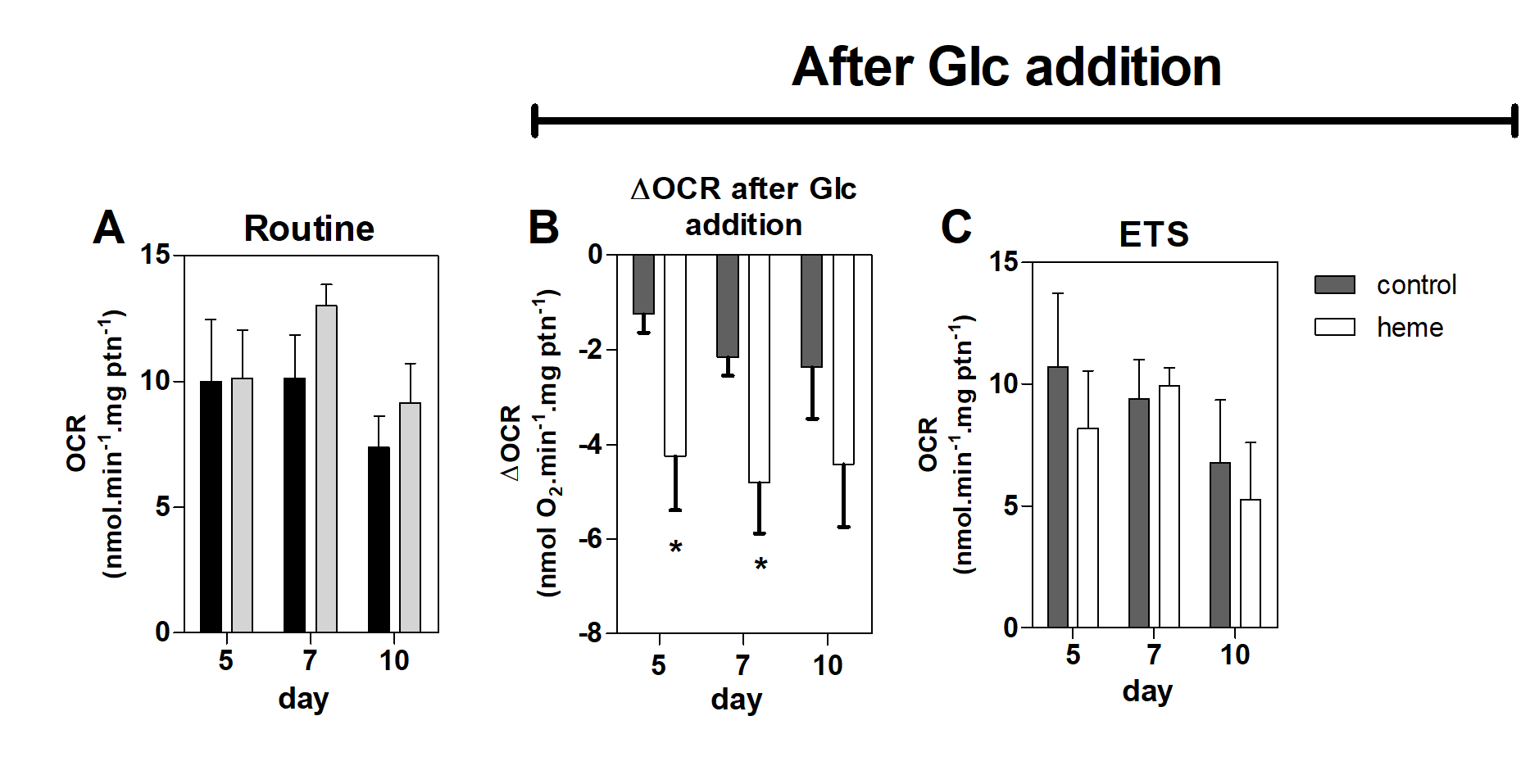

Supplement: S1 Fig — T. cruzi epimastigotes were grown in medium with or without 30 μM heme at 28°C for 5, 7, and 10 days and oxygen consumption rates (OCRs) of epimastigotes (5 × 10^7^ parasites/chamber) were evaluated by high-resolution respirometry. (A) Routine OCR, (B) Crabtree effect obtained by the difference between respiration after Glc addition (20 mM) and routine OCR (ΔOCR); and (C) maximal respiration (electron transport system, ETS) stimulated by increasing concentrations of FCCP. The results show the mean ± standard error of at least four independent experiments. *p<0.05 compared to control parasites analyzed by unpaired Student’s t-test. (TIF) [file pntd.0011725.s001.tif]

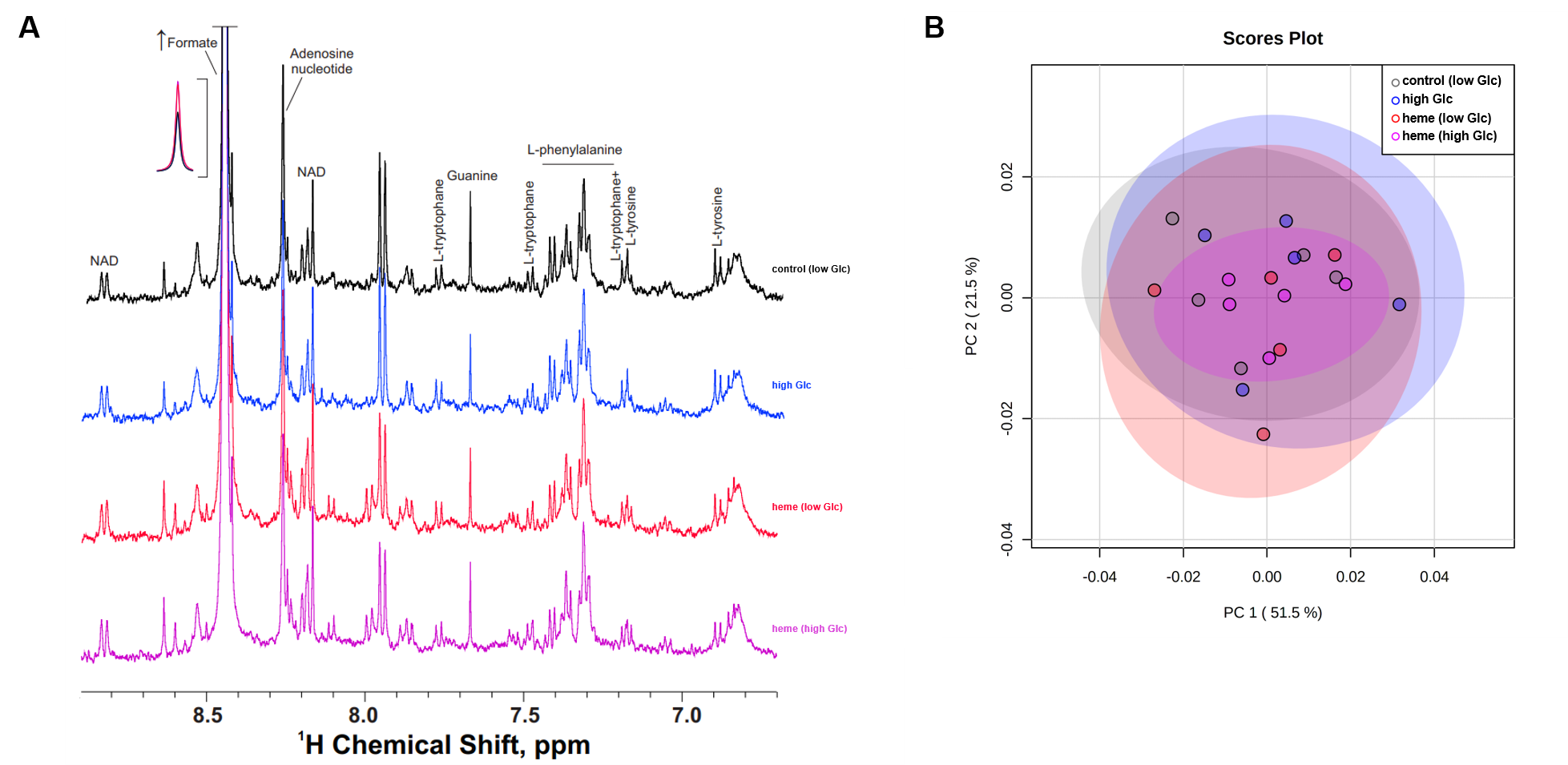

Supplement: S2 Fig — (A) Representative 1H-NMR spectrum of intracellular metabolites in the aromatic region of epimastigotes cultured in four conditions. (B) Multivariate analysis of the metabolome of four epimastigote conditions evaluated in the study. 2D-principal component analysis (PCA) score plot of the epimastigote sample extract. The figure was generated using MetaboAnalyst 5.0 software. (TIF) [file pntd.0011725.s002.tif]

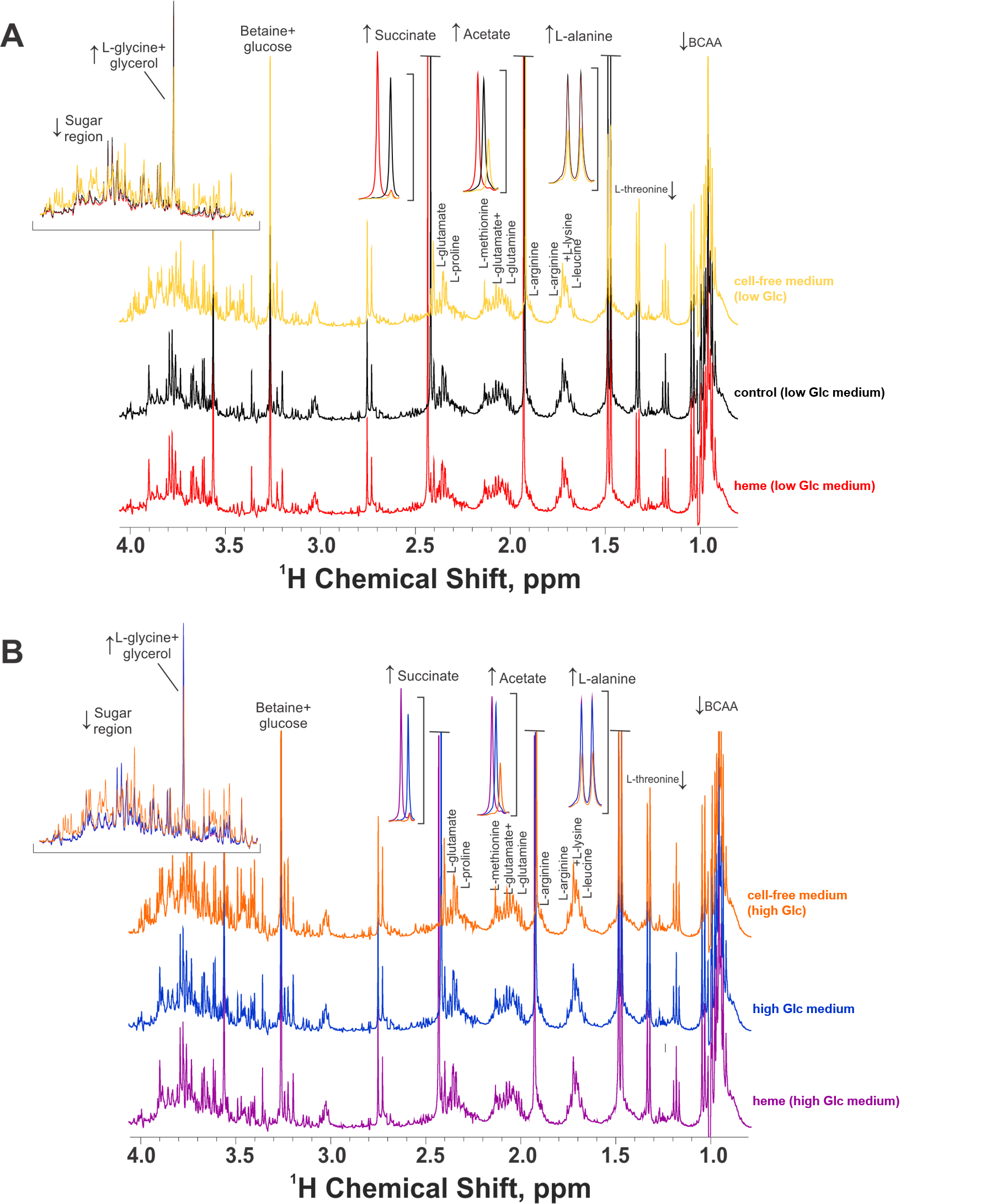

Supplement: S3 Fig — Epimastigotes were cultured for 7 days vs. (A) cell-free medium with low Glc [BHI supplemented with 10% fetal bovine serum (FBS); 6.6 mM Glc], yellow line or (B) cell-free medium with high Glc (12 mM Glc), orange line. (TIF) [file pntd.0011725.s003.tif]
